# Systematic review and meta-analysis: *de novo* combination of nucleos(t)ide analogs and pegylated interferon alpha versus pegylated interferon alpha monotherapy for the functional cure of chronic hepatitis B

**DOI:** 10.3389/fphar.2024.1403805

**Published:** 2024-07-05

**Authors:** Na Wei, Bin Zheng, Hongfu Cai, Na Li, Jing Yang, Maobai Liu

**Affiliations:** Department of Pharmacy, Fujian Medical Union Hospital, Fuzhou, China

**Keywords:** chronic hepatitis B, de novo combination, pegylated interferon alpha, nucleos(t)ide analogs, functional cure chronic hepatitis B, functional cure

## Abstract

**Introduction:** Chronic hepatitis B (CHB) is a worldwide infectious disease caused by hepatitis B virus (HBV). Optimizing antiviral treatment strategies could improve the functional cure (FC) rate of patients with CHB. This study aims to systematically review the FC rate of the *de novo* combination of nucleos(t)ide analogs (NAs) and pegylated interferon α (PEG-IFNα) versus that of PEG-IFNα monotherapy for CHB.

**Methods:** Databases were searched until 31 December 2023. Selected studies included randomized controlled trials on the *de novo* combination of NAs and PEG-IFNα versus PEG-IFNα monotherapy for 48 weeks in patients with CHB to achieve FC, which was defined as hepatitis B surface antigen (HBsAg) loss and/or HBsAg seroconversion. Meta-analysis was conducted in accordance with the efficacy at the end of treatment and different time points during follow-up.

**Results:** A total of 10 studies, encompassing 2,339 patients in total, were included. Subgroup analysis was conducted in accordance with whether first-line NAs were used. It found no statistically significant difference between HBsAg loss and HBsAg seroconversion at the end of treatment. Serum HBV DNA <500 copies/mL significantly differed between the two groups at the end of treatment and did not significantly differ during follow-up. Meanwhile, HBsAg loss and HBsAg seroconversion showed statistically significant differences at 24 weeks of follow-up. By contrast, no statistically significant difference was found in HBsAg loss at 48 weeks of follow-up.

**Discussion:** Without distinguishing the eligible preponderant population, the efficacy of the *de novo* combination of NAs and PEG-IFNα in treating patients with CHB was not superior to that of PEG-IFNα monotherapy.

**Systematic Review Registration:** PROSPERO, identifier CRD42022325239.

## 1 Introduction

Chronic hepatitis B (CHB) is a worldwide infectious disease caused by hepatitis B virus (HBV). Approximately 400 million people in the world are infected with HBV. CHB could develop into cirrhosis, liver failure, or hepatocellular carcinoma, which seriously threaten human health (Lozano et al., 2012). Persistent and active HBV replication is the most important factor leading to liver inflammation, liver fibrosis, cirrhosis, and even liver cancer (Cho et al., 2011; Shin et al., 2011). Therefore, exploring effective antiviral therapy is of great importance (Niro et al., 2013). The currently recommended antiviral drugs for HBV mainly include nucleos(t)ide analogs (NAs) and interferon α (IFNα) or pegylated interferon α (PEG-IFNα). Given that achieving complete cure is difficult, the ideal treatment goal recommended by the recent APASL guideline (KAO et al., 2021) is to achieve the functional cure (FC) of patients with CHB (Sarin et al., 2016; European Association for the Study of the Liver European Association for the Study of the Liver, 2017; Terrault et al., 2018). FC is defined as the continuous loss of hepatitis B surface antigen (HBsAg) with or without HBsAg seroconversion and undetectable serum HBV DNA after a certain course of treatment (Cornberg et al., 2020). The current suggestion, in combination with the relevant guidelines and consensus (Ning et al., 2019), is that among some patients with CHB receiving NA treatment, the eligible preponderant population (Buster et al., 2009) should receive combined treatment with PEG-IFNα. Combined therapy could improve the FC rate clinically. However, no relevant suggestions have been made on whether the combination of other nondominant groups could improve the FC rate. Therefore, whether patients with CHB receiving *de novo* combination achieved higher FC rates than those receiving PEG-IFNα monotherapy is unclear. Herein, “*de novo*” is defined as a combination of the simultaneous initiation of NAs and PEG-IFNα in naive patients. This study aims to estimate whether the *de novo* combination of NAs and PEG-IFNα is superior to PEG-IFNα monotherapy in terms of FC.

## 2 Methods

### 2.1 Search strategy

Two researchers (Na Wei and Hongfu Cai) searched the Cochrane Library, PubMed, and Embase databases. The time for retrieval was limited to the establishment of the database to 31 December 2023. Research references were included in manual retrieval to supplement relevant studies. The search was conducted by combining free words with MeSH terms. The English search words were chronic hepatitis B, CHB, pegylated interferon alpha, peginterferon α, peg-IFNα, entecavir (ETV), ETV, tenofovir dipivoxil fumarate (TDF), TDF, tenofovir alafenamide (TAF), TAF, tenofovir amibufenamide (TMF), TMF, lamivudine (LAM), LAM, adefovir (ADV), ADV, telbivudine (LdT), and LdT.

### 2.2 Inclusion and exclusion criteria

The inclusion criteria were as follows: (1) Study design: randomized controlled trial (RCT). (2) Study population: patients with CHB. (3) Intervention: comparison of the *de novo* combination of NAs and PEG-IFNα versus PEG-IFNα monotherapy. NAs were divided into first-line and non-first-line NAs for subgroup analysis. (4) Results: undetectable serum HBV DNA, HBsAg loss, or HBsAg seroconversion. (5) PEG-IFNα course of treatment: 48 weeks (Hsu et al., 2018).

The exclusion criteria were as follows: (1) non-English literature; (2) non-RCT; (3) incomplete data or inability to obtain full-text literature; (4) repeated publication of literature; (5) PEG-IFNα monotherapy compared with NA monotherapy and other types of studies on liver diseases; (6) complicated with other types of viral hepatitis, HIV, liver cirrhosis, and liver cancer; and (7) patients who have used Chinese herbal medicine and immunosuppressants or underwent liver transplantation in the past 6 months.

### 2.3 Data extraction

The two researchers independently extracted data from the study. Data included the first author, publication year, patient number, country, study design, data source/study period, therapy period, follow-up period, and therapy regimen. The protocol was registered in PROSPERO (CRD42022325239).

### 2.4 Risk of bias assessment

The included literature was all RCT studies. The methodological quality of the included studies was evaluated in accordance with the Cochrane Risk of Bias version two tool. The tool included five domains: (1) bias due to randomization, (2) bias due to deviation from the established intervention, (3) bias due to missing data, (4) bias due to outcome measurement, and (5) bias due to selective reporting. The risk of bias for each domain was classified into three levels: “low risk,” “some concerns,” and “high risk.” The overall risk of bias was determined on the basis of the level of risk of bias of each domain. If each dimension was “low risk,” then the result was denoted as “low risk.” In cases of “some concerns” and no “high risk,” the result was considered as “some concerns.” If one item had “high risk,” then the result was considered as “high risk."

### 2.5 Statistical analysis

The study aims to explore the FC rate of the *de novo* combination of NAs and PEG-IFNα versus that of PEG-IFNα monotherapy. HBsAg loss and HBsAg seroconversion were selected as the primary outcomes, and serum HBV DNA< 500 copies/mL (data sourced from hospitals) was used as the secondary outcome. The odds ratio and 95% confidence interval were calculated. The heterogeneity among the included research results was identified through a combination of chi-squared and *I* ^2^ tests of forest plots. *p* values were generated by using the chi-squared test. If no statistical heterogeneity was found among studies (*p* ≧ 0.10 and *I* ^*2*^ ≦ 50%), then fixed effect models were used. If statistical heterogeneity was found among the studies, the source of heterogeneity required further analysis. Clinical heterogeneity was treated by subgroup, sensitivity, or descriptive analysis. Random effect models were used after the influence of obvious clinical heterogeneity was excluded. Forest plots were plotted by using Review Manager (RevMan 5.3; The Cochrane Collaboration, Oxford, United Kingdom). Stata version 13.0 (Stata Corp, College Station, TX, the United States) was used for publication bias and sensitivity analyses. The analysis results determined whether a significant difference existed on the basis of the *p*-value, and *p* < 0.05 was considered to indicate difference.

## 3 Results

### 3.1 Search results and study characteristics

**FIGURE 1:**
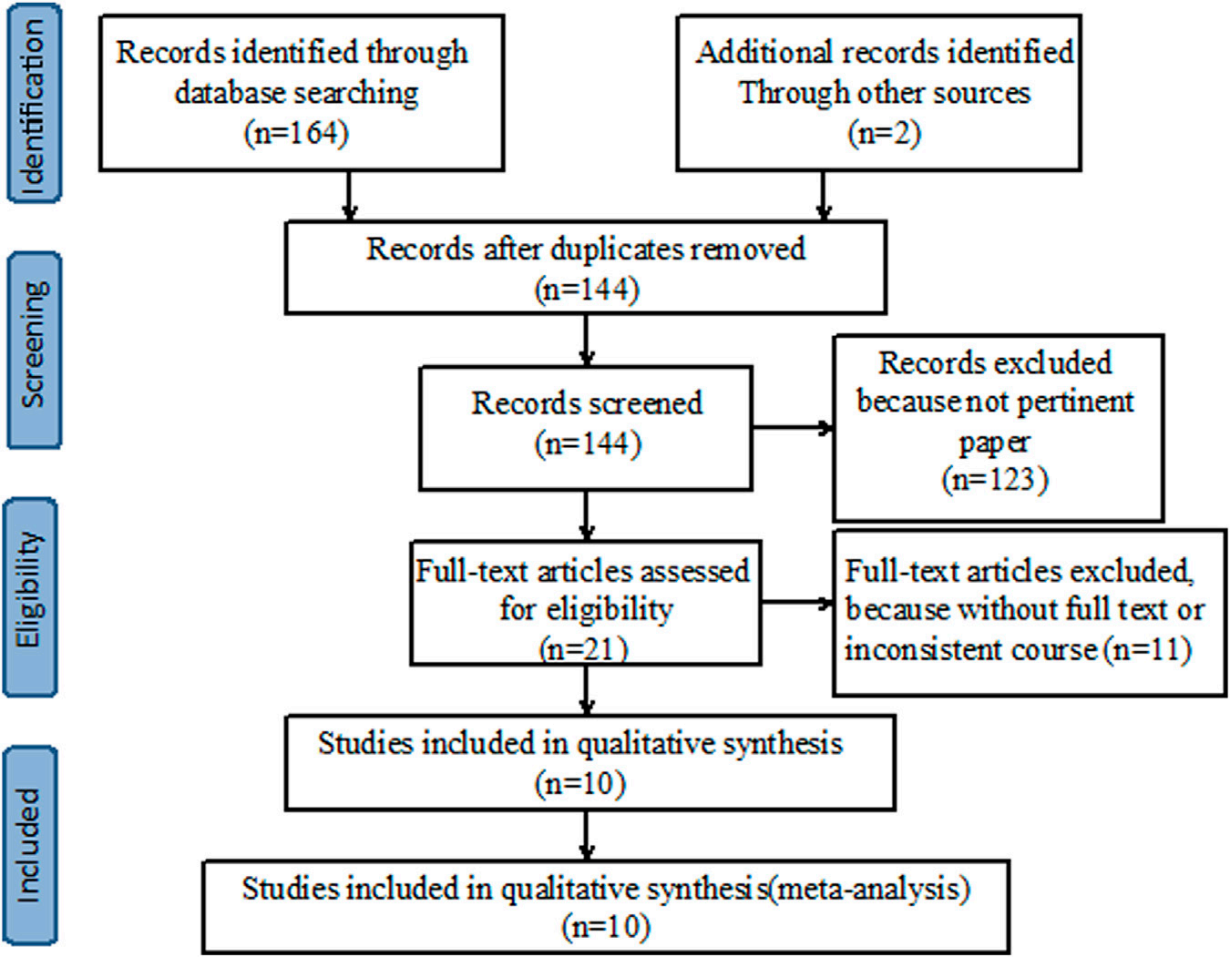
PRISMA diagram of screening and results.

A preliminary search was conducted on 166 relevant studies. A total of 22 duplicates were excluded, and 21 articles were read in full after irrelevant articles were excluded. Finally, 10 articles satisfied the inclusion criteria (Figure 1). Ten studies, with 2,339 patients in total, were included. All included studies provided undetectable HBV DNA with monotherapy and *de novo* combination therapy (Marcellin et al., 2004; Lau et al., 2005; Kaymakoglu et al., 2007; Marcellin et al., 2009; Piccolo et al., 2009; Marcellin et al., 2016; Tangkijvanich et al., 2016; Zhang et al., 2016; Ahn et al., 2018; Bahardoust et al., 2020), seven studies analyzed HBsAg loss (Marcellin et al., 2004; Marcellin et al., 2009; Piccolo et al., 2009; Marcellin et al., 2016; Tangkijvanich et al., 2016; Zhang et al., 2016; Ahn et al., 2018) and six studies explored HBsAg seroconversion (Marcellin et al., 2004; Lau et al., 2005; Piccolo et al., 2009; Marcellin et al., 2016; Tangkijvanich et al., 2016; Ahn et al., 2018). Among these studies, five were considered “low risk” and the remaining five were categorized as having “some concerns” (Table 1).

**TABLE 1:** Basic information of the included literature.

	Study	Number of patients	Country	Study design	Data source/study period	Therapy period	Follow-up period	Therapy regimen	Risk of bias
1	Marcellin, P.2016 Marcellin et al. (2016)	371	19 countries	An open-label, active-controlled randomized, multinational, superiority study	Clinical Trials. Gov number NCT 01277601 March 2011-March 2013	48w	24w	1. PEG-IFNα-2a (180μg/w)+TDF (300 mg/d) (*n* = 186) 2. PEG-IFNα-2a (180μg/w) (*n* = 185)	low risk
2	Marcellin P.2009 Marcellin et al. (2009)	356 HBeAg(−)CHB patients	United Kingdom	The initial RCT study	In the initial study (WV16241), 42 centers participated in the long-term study	48w	3y	1. PEG-IFNα-2a (180μg/w)+placebo (*n* = 177) 2. PEG-IFNα-2a (180μg/w)+LAM(100 mg/d) (*n* = 179)	some concer-ns
3	Tangkijvanich P.2016 Tangkijvanich et al. (2016)	126 HBeAg (−)CHB treatment-naïve patients	Thailand	This investigator-initiated, prospective, randomized, open-label study	Study was conducted in King Chulalongkorn Memorial Hospital, Bangkok, Thailand, between November 2010 and April 2014	48w	48w	1. PEG-IFNα-2b (1.5 μg/kg/week) (*n* = 63) 2. PEG-IFNα-2b (1.5 μg/kg/week) +ETV (0.5 mg/d) (n = 63)	some concer-ns
4	Ahn S H 2018 Ahn et al. (2018)	371 CHB patients	15 countries	A randomized open-label active-controlled, multinational, superiority trial (NCT01277601)	An analysis of data from study GS-US-174–0,149	48w	24w and 72w	1. PEG-IFNα-2a (180μg/w)+TDF (300 mg/d) (n = 186) 2. PEG-IFNα-2a (180μg/w) (n = 185)	some concer-ns
5	Bahardoust M 2020 Bahardoust et al. (2020)	49 HBeAg(−)CHB patients	Iran	This randomized, open-label and single center study	Study was conducted in Rasoul e Akram Hosptial from October 2014 to October 2017(Tehran, Iran)	48w	—	1. PEG-IFNα (180μg/w)+TDF (300 mg/d) (n = 25) 2. PEG-IFNα-2a (180μg/w) (n = 24)	low risk
6	Kaymakoglu S 2007 Kaymakoglu et al. (2007)	48 HBeAg(−)CHB patients	Turkey	Prospective, open-label, randomized study	Study was conducted in eight teriary hospitals between June 2001 and January 2004	48w	24w	1. PEG-IFNα-2b (1.5 μg/kg/week) (n = 19) 2. PEG-IFNα-2b (1.5 μg/kg/week) + LAM(100 mg/d) (n = 29)	some concer-ns
7	Lau G K K 2005 Lau et al. (2005)	542 HBeAg(+)CHB patients	16 countries in Asia, Australasia, Europe, and North and South America	This multicenter, randomized, partially double-blind study	Study was conducted at 67 sites in 16 countries	48w	24w	1. PEG-IFNα-2a (180μg/w)+placebo (n = 271) 2. PEG-IFNα-2a (180μg/w)+LAM(100 mg/d) (n = 271)	low risk
8	Marcellin P 2004 Marcellin et al. (2004)	356 HBeAg(−)CHB patients	13 countries principally in Asia and Europe	This multicenter, randomized, partially double-blind study	Study was conducted at 54 sites in 13 countries	48w	24w	1. PEG-IFNα-2a (180μg/w)+placebo (n = 177) 2. PEG-IFNα-2a (180μg/w)+LAM(100 mg/d) (n = 179)	low risk
9	Zhang K 2016 Zhang et al. (2016)	65 HBeAg(+)CHB patients	China	This investigator-initiated, prospective, randomized, open-label study	Study was conducted in the Department of Infectious Diseases of the Third Affiliated Hospital of Sun Yat-sen University between October 2009 and December 2014	48w	48w	1. PEG-IFNα-2a (135μg/w) (n = 32) 2. PEG-IFNα-2a (135μg/w)+ADV (10 mg/d) (n = 33)	low risk
10	Piccolo P 2009 Piccolo et al. (2009)	60 HBeAg(−)CHB patients	In central Italy	A multicenter, randomized controlled trial	Study was conducted in eight outpatient hepatology/infectious disease clinics in central Italy	48w	8w and 24w	1. PEG-IFNα-2a (180μg/w) (*n* = 30) 2. PEG-IFNα-2a (180μg/w)+ADV (10 mg/d) (*n* = 30)	some concer-ns

### 3.2 Serum HBV DNA< 500 copies/mL, HBsAg loss, and HBsAg seroconversion

**FIGURE 2:**
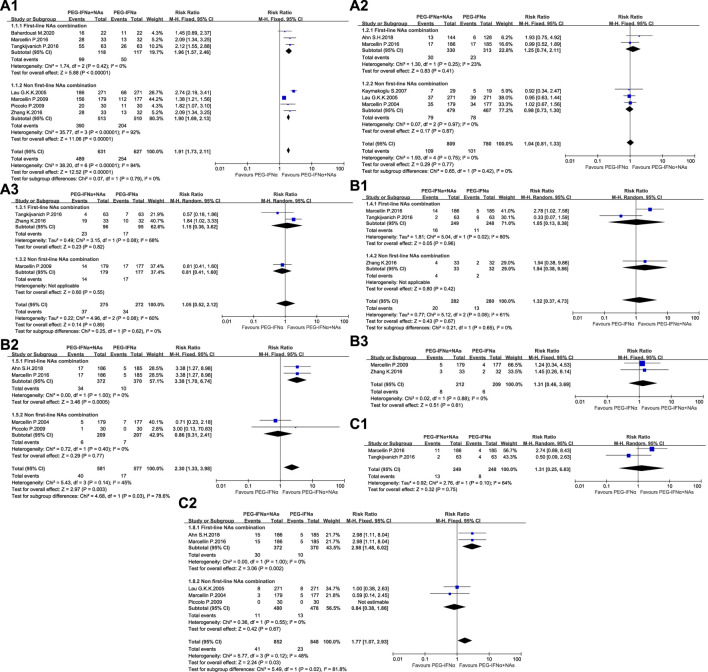
Meta-analysis of OR and 95% CI for serum HBV DNA< 500 copies/mL **(A)**, HBsAg loss **(B)**, and HBsAg seroconversion **(C)** 1. At the end of treatment, 2. At 24 weeks of follow-up, and 3. At 48 weeks of follow-up.

The antiviral efficacy of ETV, TDF, and TAF was significantly better than that of LAM, ADV, and LdT. Subgroup analysis was conducted in accordance with the antiviral efficacy of NAs to reduce clinical heterogeneity and avoid masking potential differences between two groups. ETV, TDF, TAF, and TMF are first-line NAs, whereas LAM, ADV, and LdT are non-first-line NAs. Analysis was conducted in accordance with the efficacy at the end of treatment and different time-points of follow-up to minimize heterogeneity. The meta-analysis results are shown in Table 2 and Figure 2.

**TABLE 2:** Effect size of meta-analysis results.

Effect indicators	OR [95%CI], P		
	End of treatment (48 W)	24 W after treatment (72 W)	48 W after treatment (96 W)
Serum HBV DNA<500copies/mL	5.67 [4.37, 7.34], *p* < 0.00001	1.04 [0.78, 1.40], *p* = 0.77	1.11 [0.43, 2.90], *p* = 0.83
HBsAg loss	1.34 [0.34, 5.25], *p* = 0.67	2.39 [1.35, 4.23], *p* = 0.003	1.33 [0.45, 3.91], *p* = 0.61
HBsAg seroconversion	1.32 [0.24, 7.40], *p* = 0.75	1.81 [1.08, 3.05], *p* = 0.02	—

### 3.3 Publication bias

**FIGURE 3:**
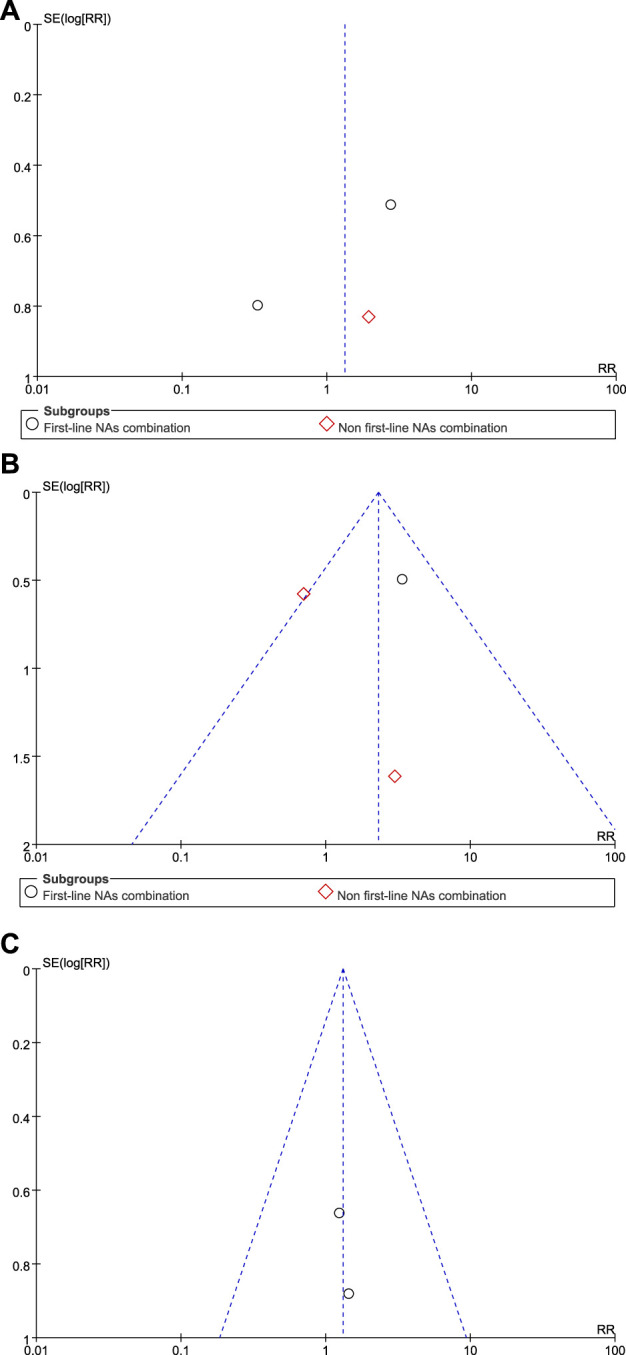
Funnel plot analyses of HBsAg loss **(A)** at the end of treatment **(B)** at 24 weeks of follow-up, and **(C)** at 48 weeks of follow-up.

**FIGURE 4:**
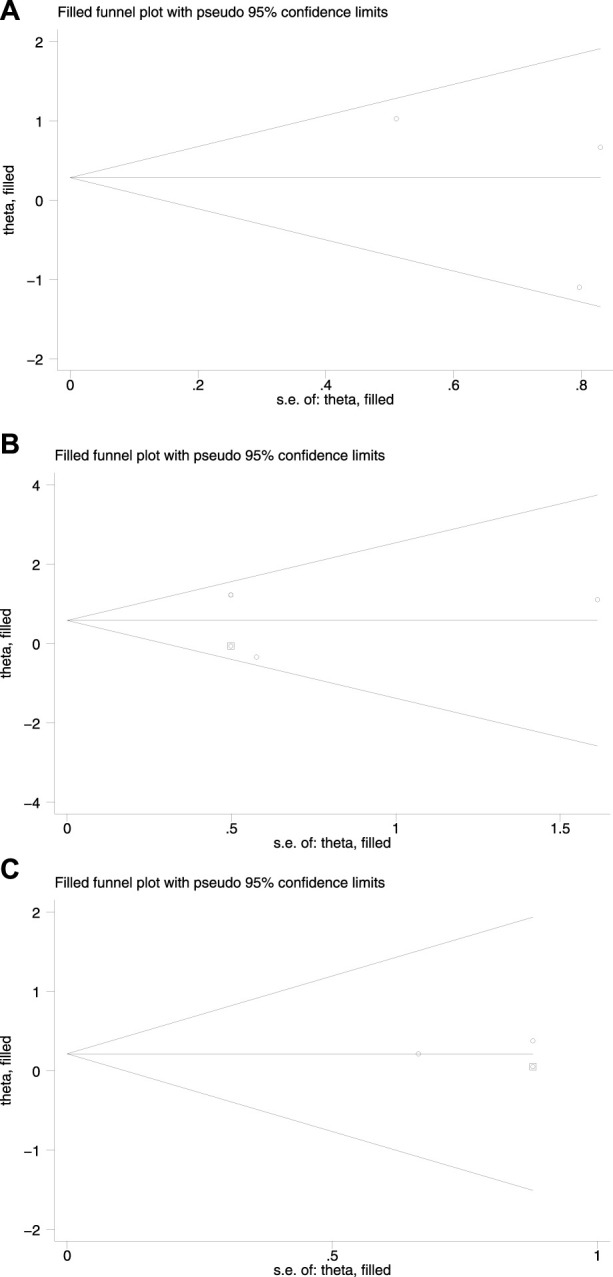
Redrawn funnel plot analyses of HBsAg loss **(A)** at the end of treatment **(B)** at 24 weeks of follow-up, and **(C)** at 48 weeks of follow-up.

The funnel plot showed that the effect values of the study were relatively scattered, indicating that the sample size of the studies on HBsAg loss was small (Figure 3). Egger’s test revealed that HBsAg loss had no significant publication bias. Begg’s test showed no significant publication bias in HBsAg loss. The funnel plot obtained became symmetrical after being clipped through the trim-and-fill method (Figure 4).

### 3.4 Sensitivity analyses

**FIGURE 5:**
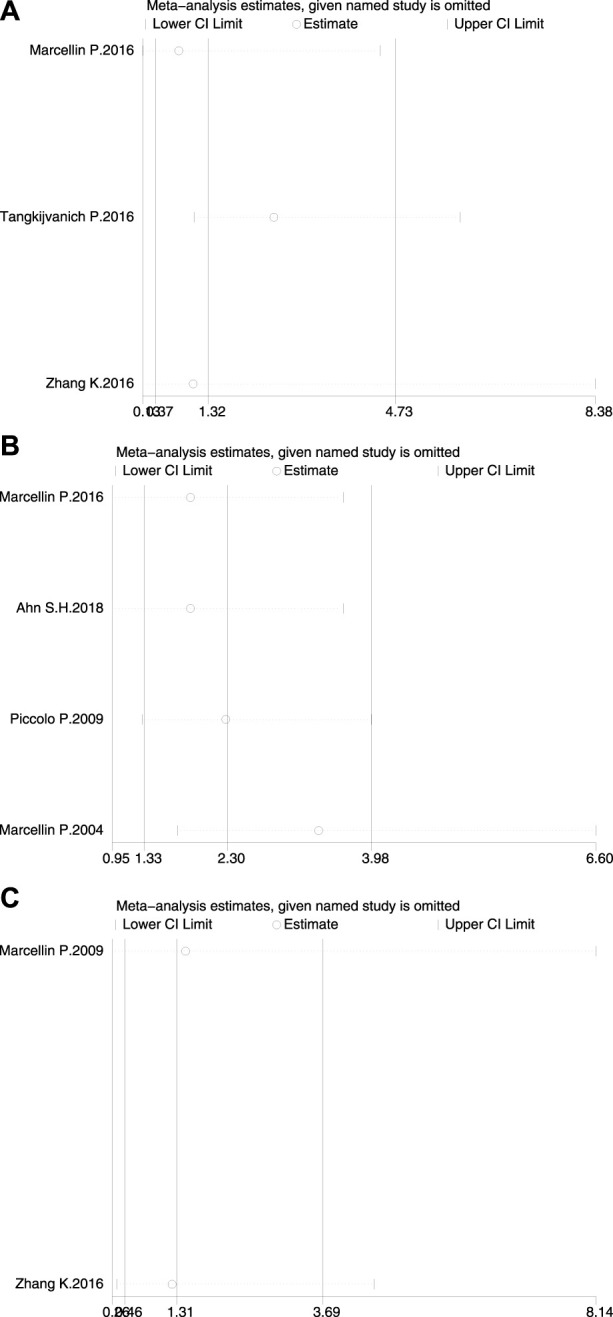
Sensitivity analysis of HBsAg loss **(A)** at the end of treatment **(B)** at 24 weeks of follow-up, and **(C)** at 48 weeks of follow-up.

Sensitivity analyses were performed on the effect of the results by deleting one study on HBsAg loss at a time (Figure 5). None of the studies had a significantly sufficient effect on the combined OR, thus confirming that the combined results were robust and reliable.

## 4 Discussion

NAs and Peg-IFNα are the two main types of antiviral drugs for CHB. The mechanisms of their antiviral effects differ. NAs could inhibit HBV DNA polymerase competitively, which could then inhibit viral replication effectively and ameliorate liver inflammation. Meanwhile, Peg-IFNα could inhibit viral replication and transcription by enhancing the function of HBV-specific T lymphocytes and producing various antiviral proteins through the interferon signal pathway to exert an antiviral effect and enhance the immune function of the body (Chelbi-Alix and Wietzerbin, 2007). Although no antiviral drugs with increased effectiveness have been developed (Wong et al., 2022), antiviral efficacy and FC rate could be improved by optimizing the existing antiviral treatment scheme.

*De novo* combination therapy is gradually and increasingly being applied in clinical practice. A previous meta-analysis revealed that PEG-IFNα and LAM combination therapy was not superior to PEG-IFNα monotherapy in terms of HBsAg clearance and serological conversion. LAM was selected in the present study, but its drug resistance rate was found to be high. Song et al. (2021) studied patients with CHB, low baseline HBsAg levels, and long PEG-IFNα treatment courses and found that these patients could easily achieve FC. However, no relevant research was conducted on patients with CHB and high baseline levels. Liu et al. (2020) compared the efficacy of *de novo* combination therapy with that of monotherapy at the end of treatment. They combined RCT and non-RCT studies and discovered no statistical difference in HBsAg loss between the two treatments. However, due to the lack of follow-up data, no study was conducted after treatment, and the combined results of different study types exhibited heterogeneity. The present study mainly selected RCTs on the *de novo* combination of NAs and PEG IFNα versus PEG-IFNα monotherapy for patients with CHB to achieve FC after generating virological and serological responses. The results showed a statistically significant difference in serum HBV DNA <500 copies/mL between the two groups at the end of treatment. Meanwhile, the two schemes showed no difference in HBsAg loss and seroconversion at the end of treatment. The difference in the results for serum HBV DNA <500 copies/mL was not statistically significant during follow-up. The results of HBsAg loss and seroconversion demonstrated statistically significant differences at 24 weeks of follow-up. By contrast, no statistically significant difference in HBsAg loss was found at 48 weeks of follow-up. The results of HBsAg loss and seroconversion at 24 weeks of follow-up were consistent. Marcellin et al. (2009) discovered no statistically significant differences in HBsAg loss between the two treatment schemes at 144 weeks of follow-up. However, HBsAg seroconversion lacked follow-up data exceeding 24 weeks. PEG-IFNα monotherapy could be considered for the initial treatment of patients with CHB without contraindications for IFNα use. But during the period of immunosuppressive therapy, NAs with rapid efficacy and low incidence of drug resistance, such as ETV, TDF, and TAF, rather than IFNα, should be selected to prevent HBV reactivation (Spera, 2022).

This systematic review has limitations. Among the 10 studies included, some on the combined analyses of HBeAg-positive and -negative patients with CHB did not mention the baseline level of HBsAg; however, the level of HBsAg is closely related to FC (Wen et al., 2024). Some studies mentioned the baseline level of HBsAg, which was approximately 10^4^ IU/mL. Given that the lower limit of HBV DNA detection at our hospital is 500 copies/mL, we included studies with results of HBV DNA <500 copies/mL. However, the lower detection limit of HBV DNA at every hospital differs. Among NA combinations (ETV, LAM, ADV, TDF, and TAF), LAM has a high drug resistance rate () and TAF shows good virological suppression (He et al., 2023). Although subgroup analysis was conducted, the combination of the above factors may have led to differences among studies, which may affect the reliability of this systematic review. Most of the included RCT studies lacked a detailed description of the hidden randomization scheme. Given this situation, the possibility of implementation and selective biases could not be ruled out. The purpose of this study is to investigate whether the addition of NAs enhances antiviral efficacy on the basis of a 48-week course of PEG-IFNα application as a course of treatment. For patients with CHB, NAs alone require a relatively long-term course of treatment. In terms of therapeutic efficacy, the difference in HBsAg loss between the two groups at the end of treatment (48 weeks) was not statistically significant. However, at 24 weeks of follow-up, the combined treatment was superior to monotherapy. At 48 weeks of follow-up, no statistically significant difference was observed between the two schemes. One related study (Marcellin et al., 2009) had a follow-up of 144 weeks and found that combination therapy was not superior to monotherapy. However, no studies with long follow-ups have been reported given that long follow-up data for HBsAg seroconversion, which could only be inferred from follow-up data on HBsAg loss, are unavailable. The present research results have shown that combination therapy is not superior to monotherapy. However, this systematic review only compared the FC rates of *de novo* combination and PEG-IFNα monotherapy. The FC rate is closely related to the baseline level of HBsAg and whether HBsAg drops rapidly (Siederdissen and Cornberg, 2014; Martinot-Peignoux et al., 2014; Wu et al., 2015; Tang et al., 2023). The problem of the best administration time and scheme for combined treatment require further exploration. Therefore, research with a large sample size is still needed, and the possible influencing factors should be included for comprehensive evaluation. The present research provides information for similar treatment schemes for CHB in China and other countries.

## 5 Conclusion

This meta-analysis revealed that the FC rates of the *de novo* combination of NAs and PEG-IFNα in the treatment of patients with CHB were not superior to those of PEG-IFNα monotherapy excluding the eligible preponderant population. The use of PEG-IFNα monotherapy for patients with CHB who have indications for PEG-IFNα treatment is recommended.

## Data Availability

The datasets presented in this study can be found in online repositories. The names of the repository/repositories and accession number(s) can be found in the article/supplementary material.
